# Formal preparation of regioregular and alternating thiophene–thiophene copolymers bearing different substituents

**DOI:** 10.3762/bjoc.16.31

**Published:** 2020-03-05

**Authors:** Atsunori Mori, Keisuke Fujita, Chihiro Kubota, Toyoko Suzuki, Kentaro Okano, Takuya Matsumoto, Takashi Nishino, Masaki Horie

**Affiliations:** 1 Department of Chemical Science and Engineering, Kobe University, 1-1 Rokkodai, Nada, Kobe 657-8501, Japan; 2 Research Center for Membrane and Film Technology, Kobe University, 1-1 Rokkodai, Nada, Kobe 657-8501, Japan; 3 Department of Chemical Engineering, National Tsing Hua University, 101, Sec. 2, Kuang-Fu Road, Hsinchu 30013, Taiwan

**Keywords:** alternating copolymer, nickel(II) catalyst, oligosiloxane, regioregular polythiophene, solubility

## Abstract

Differently substituted thiophene–thiophene-alternating copolymers were formally synthesized employing a halo-bithiophene as a monomer. Nickel-catalyzed polymerization of bithiophene with substituents at the 3-position, including alkyl-, fluoroalkyl-, or oligosiloxane-containing groups, afforded the corresponding copolymers in good to excellent yield. The solubility test in organic solvents was performed to reveal that several copolymers showed a superior solubility. X-ray diffraction analysis of the thin film of the alternating copolymers composed of methyl and branched oligosiloxane substituents was also performed, and the results suggested the formation of a dual-layered film structure.

## Introduction

**Scheme 1:**
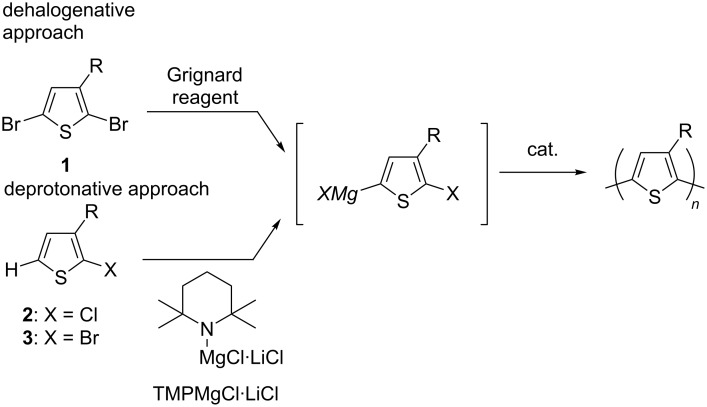
Cross-coupling polymerization of thiophene.

Polythiophenes attract much attention in materials science because of their extended π-conjugation, which is applied for a wide range of electronic materials. In particular, the regioregular polymers with a head-to-tail (HT) orientation with respect to the substituent at the 3-position are extensively studied to date since they generally show superior performances as materials [1–6]. Cross-coupling polymerization catalyzed by a transition metal complex has been recognized as an effective tool to afford the regioregular polythiophene in which 2,5-dihalo-3-substituted thiophene **1** is employed as a monomer precursor, converting to the corresponding organometallic monomer by a halogen−magnesium exchange reaction with a Grignard reagent. The employment of **1** leading to polythiophene has been shown to proceed in a dehalogenative manner [3]. We have recently shown that the generation of the organometallic monomer species can alternatively also be achieved by deprotonation, using 2-halo-3-substituted thiophene **2** or **3** with a bulky magnesium amide Knochel–Hauser base (TMPMgCl⋅LiCl) [7], followed by polymerization catalyzed by a nickel complex, leading to the regioregular HT polythiophene (Scheme 1) [8–9]. An additional feature of the deprotonative protocol for polythiophene is the possibility to use chlorothiophene **3**, in which the use of a nickel N-heterocyclic carbene (NHC) complex was found to be effective [10–11]. We have also engaged in the design of the side chain of polythiophenes, and several functionalities have been successfully introduced [12–14]. We further focused on the copolymerization of thiophene, employing differently substituted thiophene monomers, with which several copolymerizations are plausible, giving thiophene–thiophene copolymers of random (statistical) [15], gradient [16–17], block [18–19], alternating [20–23], etc. [24–25] makeup. We are thus interested in the preparation of alternating polythiophenes bearing two kinds of different substituents. We envisaged that such an alternating copolymer in perfect regularity can be achieved by deprotonative polymerization employing a bithiophene with different substituents at the 3- and 3'-position.

**Scheme 2:**
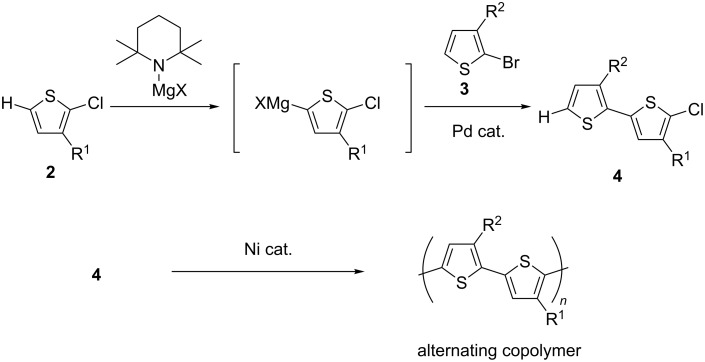
Polymerization of bithiophene.

We have recently shown that the coupling of 2-chloro-3-substituted thiophene **2** with 2-bromo-3-substituted thiophene **3** efficiently gave chlorobithiophene **4** in a facile manner (Scheme 2) [25]. The use of a palladium catalyst efficiently suppressed the undesired polymerization to afford the HT halobithiophene with different substituents [26]. Compared to an alternative pathway to **4**, in which initial coupling is followed by chlorination, this protocol exploits the improved deprotonation efficiency of **2** toward 3’-unsubstituted 3-substituted bithiophene, and this method enabled the synthesis of **4** (where R^1^ = H) regioselectively. Polymerization of **4** (where R^1^ = *n*-hexyl and R^2^ = (CH_2_)_4_Si(Me_2_)OSiMe_3_) was also examined preliminarily, and it was confirmed that the alternating copolymer was obtained with extremely high regularity. Herein, we wished to study the polymerization of bithiophene **4** possessing several kinds of substituents and a variety of functionalities. Since the homopolymer is considered rather insoluble in most organic solvents, the presumed improved solubility of the related alternating copolymers was also part of this study.

## Results and Discussion

**Scheme 3:**
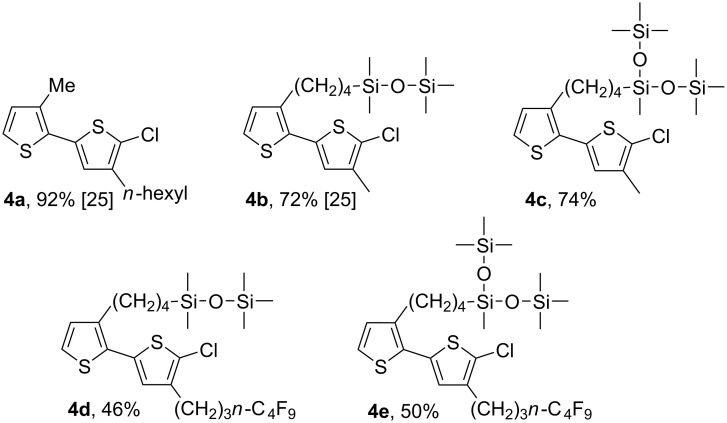
Preparation of chlorobithiophenes.

The synthesis of chlorobithiophenes with different substituents at the 3- and 3'-position was carried out in a manner that we have described previously [25]. We chose five chlorobithiophenes as monomer precursors for the alternating copolymers, as summarized in Scheme 3. The cross-couplings, as shown in Scheme 2, proceeded smoothly to afford the bithiophenes **4** in 46–92% yield.

The synthesis of the alternating copolymers was carried out with monomer precursors **4** by deprotonation with Knochel–Hauser base followed by the addition of the nickel catalyst NiCl_2_(PPh_3_)IPr to initiate the polymerization of bithiophene.

**Scheme 4:**
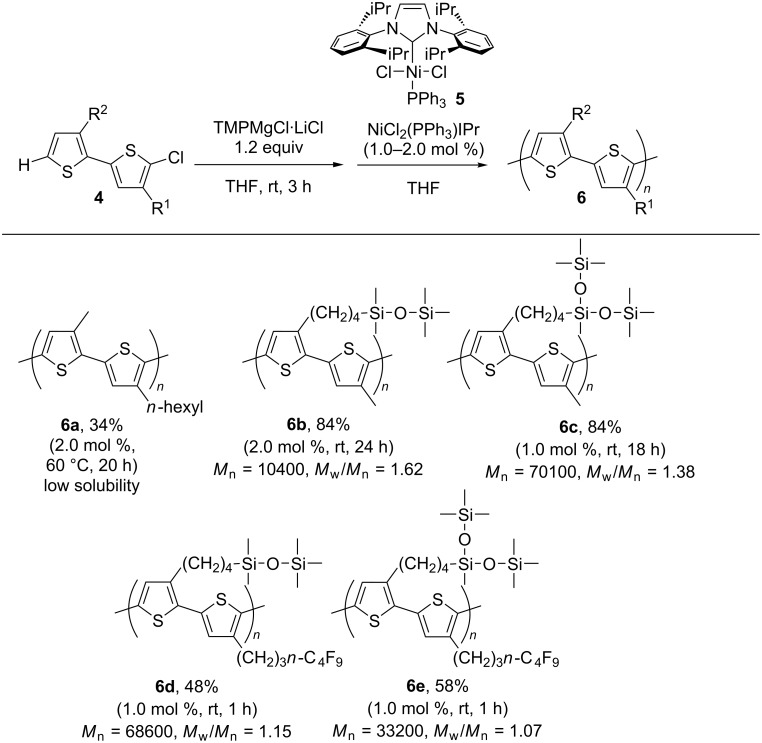
Polymerization of chlorobithiophenes.

We first carried out the polymerization of chlorobithiophene **4a**, bearing hexyl and methyl substituents at the 3- and 3'-position, respectively. Although the polymerization took place in moderate yield (34%), the formation of hardly soluble precipitates was observed during the reaction, and the thus obtained solid was found to fail to dissolve in any organic solvent. As previously reported for the regioregular polythiophene synthesis, poly(3-hexylthiophene) (P3HT) can smoothly be dissolved in several organic solvents. In contrast, there have been few reports on the preparation of regioregular polythiophene bearing a methyl group at the 3-position [27]. Accordingly, the incorporation of the alternating methyl substituent would result in much inferior solubility as compared to the alternating copolymer. Several chlorobithiophenes **4** were then similarly subjected to the polymerization. The results of the alternating copolymerizations are summarized in Scheme 4. The deprotonation by the Knochel–Hauser base was carried out at room temperature for 3 h. The addition of the nickel catalyst **5** and further stirring at room temperature followed. The reactions proceeded smoothly to afford the corresponding formally alternating copolymers in 48–84% yields [28]. The molecular weight of the products was found to be controllable based on the ratio of monomer/catalyst feed, and the molecular weight distributions were relatively narrow.

**Figure 1:**
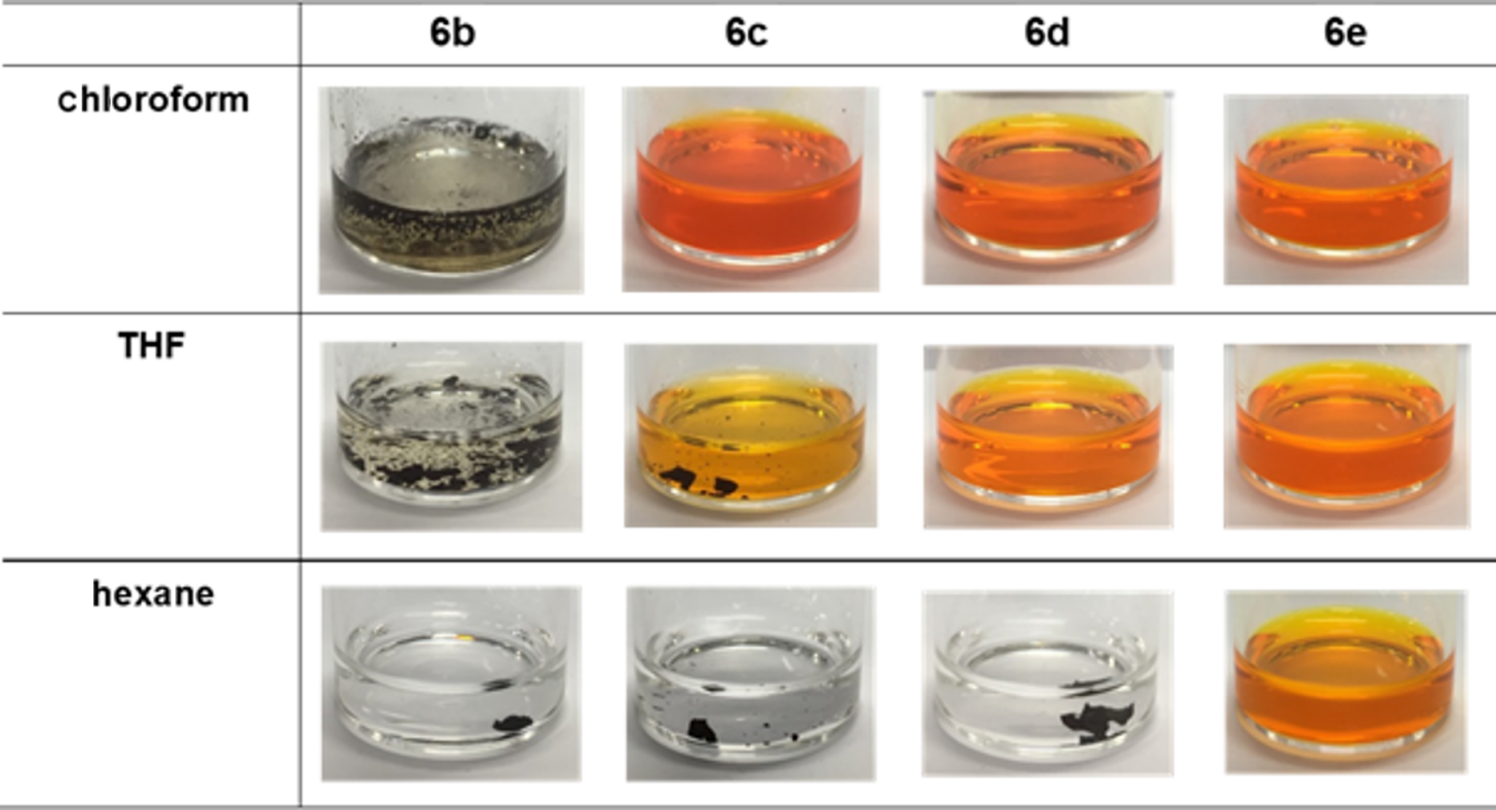
Solubility tests of alternating copolymer **6** (1 mg of material dissolved in 1 mL of the solvent).

Solubility tests of the obtained polymers were performed as summarized in Figure 1. Although the alternating copolymer **6b**, substituted with methyl and C-4 alkyl groups as well as terminal pentamethyldisiloxane groups, was obtained with a slightly low molecular weight, suggesting improved solubility compared to **6a** (R^2^ = Me and R^1^ = *n*-hexyl), the attempted dissolution of the obtained polythiophene **6b** in chloroform was unsuccessful. Switching the oligosiloxane moiety to a branched derivative (R^2^ = (CH_2_)_4_Si(Me)(OSiMe_3_)_2_) in **6c** remarkably improved the solubility, and the copolymer **6c** was soluble in chloroform, whereas the dissolution in hexane was unsuccessful. Copolymers bearing a fluoroalkyl substituent ((CH_2_)_3_*n*-C_4_F_9_), with a corresponding homopolymer solubility that was lower than that of the long-chained alkyl derivatives, were then examined. The alternating copolymer **6d**, bearing fluoroalkyl and non-branched disiloxane substituents was readily soluble in chloroform, while the attempted dissolution of **6d** in hexanes was unsuccessful. However, a remarkable solubility of the copolymer **6e** bearing a fluoroalkyl group and a branched oligosiloxane unit in hexanes was noted.

**Figure 2:**
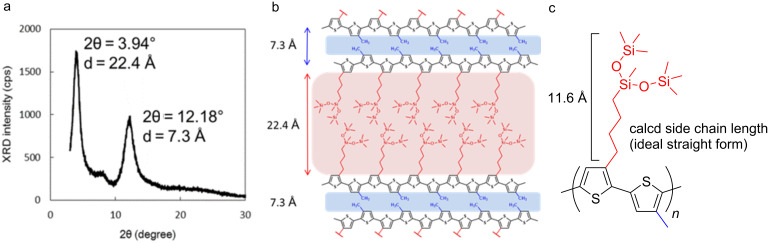
XRD measurement and prediction of the bilayer lamellar structure of polymer **6c**. a) XRD analysis. b) Suggested bilayer lamellar structure. c) Side chain length.

XRD analysis of the copolymer **6c**, bearing branched oligosiloxane and methyl groups, was carried out. Two remarkable peaks were observed at 2θ = 3.94° and 12.18°, respectively, as shown in Figure 2a. The result suggested that the thin film of the alternating copolymer **6c** had a bilayer lamellar structure with 7.3 Å and 22.4 Å distances, respectively (Figure 2b) [13,29–30]. Molecular modeling of the alternating copolymer **6c** suggested chain lengths of 11.6 Å and 2.2 Å, respectively. The values of the observed layer distances of copolymer **6c** closely corresponded to twice the values, 11.6 × 2 and 2.2 × 2 (Figure 2c), in which the conformation of the carbon–carbon single bond between the thiophene rings was *anti*. The proposed layer distance of regiorandom poly(3-methylthiophene) of 7.7 Å reported by Yan and co-workers was close to our result from the XRD analysis (7.3 Å) corresponding to the aggregation of the alternating methyl substituent [27].

## Conclusion

In summary, we showed that formally alternating thiophene–thiophene copolymers could be synthesized by employing differently substituted halobithiophenes as monomers in nickel-catalyzed deprotonative polymerizations. The introduction of oligosiloxanes as side chains improved the solubility in organic solvents, and copolymer components involving less soluble functional groups, such as short alkyl chains or fluoroalkyl groups, could be incorporated into the alternating copolymers. X-ray diffraction measurements revealed that the alternating copolymers bearing different side chain lengths had dual-layer structures in the thin-film state.

## Experimental

### General

Polymerization was carried out with the standard Schlenk technique under a nitrogen or argon atmosphere. ^1^H NMR (400 MHz), ^19^F NMR (376 MHz), and ^13^C{^1^H} NMR (100 MHz) spectra were measured on a JEOL ECZ400 spectrometer as a CDCl_3_ solution, unless otherwise noted. The chemical shifts were expressed in ppm with CHCl_3_ (7.26 ppm for ^1^H), C_6_F_6_ (−164.9 ppm for ^19^F), or CDCl_3_ (77.16 ppm for ^13^C) as internal standards. IR spectra were recorded on Bruker Alpha spectrometer with an ATR attachment (Ge). High-resolution mass spectra (HRMS) were measured using a JEOL JMS-T100LP AccuTOF LC-Plus apparatus (ESI) with a JEOL MS-5414DART attachment. For thin-layer chromatography (TLC) analyses throughout this work, Merck precoated TLC plates (silica gel 60 F_254_) were used. Purification by HPLC with preparative SEC columns (JAI-GEL-1H and JAI-GEL-2H) was performed by a JAI LC-9201 system. SEC analyses were carried out with a standard HPLC system equipped with a UV detector at 40 °C using CHCl_3_ as an eluent with Shodex KF-402HQ and KF-404HQ. Molecular weights and molecular weight distributions were estimated on the basis of the calibration curve obtained by 6 standard polystyrenes. UV–vis absorption spectra of the polymer films were measured with a Shimadzu UV-3150 spectrometer. XRD analysis was carried out with a Rigaku RINT-2000(CuKα) device. Concerning the solvent of the nickel- and palladium-catalyzed reactions, THF (anhydrous grade) was purchased from Kanto Chemical Co. Ltd. and passed through alumina and copper columns (Nikko Hansen & Co. Ltd.) or distilled from a sodium dispersion in mineral oil/benzophenone ketyl [31] prior to use. The Knochel–Hauser base (TMPMgCl⋅LiCl) was purchased from Sigma-Aldrich Co. Ltd. as a 1 M THF solution. NiCl_2_(PPh_3_)IPr (**5**) was purchased from TCI Co. Ltd. All other chemicals were purchased and used without further purification. The preparation of the chlorobithiophenes **4a** and **4b** was performed in a manner reported previously [25].

The synthesis of the chlorobithiophenes **4** was carried out in a manner shown in our previous report [25]. The spectroscopic properties and analytical data for **4** are summarized below.

**2-Chloro-3-hexyl-5-(3-methylthiophen-2-yl)thiophene (4a)** [25]**:** 92% yield as a light yellow oil. ^1^H NMR (500 MHz, CDCl_3_) δ 0.92 (t, *J* = 7.5 Hz, 3H), 1.31–1.43 (m, 6H), 1.58–1.67 (m, 2H), 2.37 (s, 3H), 2.59 (t, *J* = 7.5 Hz, 2H), 6.83 (s, 1H), 6.87 (d, *J* = 5.0 Hz, 1H), 7.14 (d, *J* = 5.0 Hz, 1H); ^13^C{^1^H} NMR (100 MHz, CDCl_3_) δ 14.2, 15.3, 22.8, 28.2, 29.1, 29.7, 31.8, 123.5, 124.0, 126.2, 130.6, 131.4, 133.1, 134.2, 139.8; IR (ATR): 2954, 2926, 2856, 1463, 1199, 1042, 830, 705, 617 cm^−1^; HRMS (DART-ESI^+^) *m*/*z*: calcd for C_15_H_20_^35^ClS_2_, 299.0695; found, 299.0687.

**2-Chloro-3-methyl-5-(3-(4-(1,1,3,3,3-pentamethyldisiloxy)butan-1-yl)thiophen-2-yl)thiophene (4b)** [25]**:** 72% yield as a light yellow oil. ^1^H NMR (400 MHz, CDCl_3_) δ 0.0 (s, 6H), 0.05 (s, 9H), 0.50–0.57 (m, 2H), 1.33–1.44 (m, 2H), 1.58–1.68 (m, 2H), 2.19 (s, 3H), 2.67–2.74 (m, 2H), 6.77 (s, 1H), 6.90 (d, *J* = 5.0 Hz, 1H), 7.15 (d, *J* = 5.0 Hz, 1H); ^13^C{^1^H} NMR (100 MHz, CDCl_3_) δ 0.5, 2.2, 13.7, 18.4, 23.3, 29.0, 34.5, 124.0, 124.6, 127.6, 128.4, 130.0, 132.7, 134.7, 140.0; IR (ATR): 2955, 2924, 2858, 1567, 1411, 1252, 1194, 1051, 840, 807, 783, 753, 687, 651, 625 cm^−1^; HRMS (DART-ESI^+^) *m/z*: calcd for C_18_H_30_^35^ClS_2_Si_2_, 417.0951; found, 417.0979.

**2-Chloro-3-methyl-5-(3-(4-(bis(trimethylsiloxy)(methyl)silyl)butan-1-yl)thiophen-2-yl)thiophene (4c)**: 74% yield as a light yellow oil. ^1^H NMR (400 MHz, CDCl_3_) δ 0.0 (s, 3H), 0.07 (s, 18H), 0.44–0.51 (m, 2H), 1.33–1.42 (m, 2H), 1.58–1.67 (m, 2H), 2.19 (s, 3H), 2.67–2.73 (m, 2H), 6.77 (s, 1H), 6.90 (d, *J* = 5.0 Hz, 1H), 7.15 (d, *J* = 5.0 Hz, 1H); ^13^C{^1^H} NMR (100 MHz, CDCl_3_) δ −0.1, 2.0, 13.7, 17.6, 23.1, 28.9, 34.4, 124.1, 124.5, 127.6, 130.0, 130.1, 132.6, 134.7, 140.0; IR (ATR): 2957, 1411, 1256, 1045, 840, 799, 783, 754, 688, 651 cm^−1^; HRMS (DART-ESI^+^) *m/z*: calcd for C_20_H_35_^35^ClO_2_S_2_Si_3_, 491.1153; found, 491.1177.

**2-Chloro-3-(4,4,5,5,6,6,7,7,7-nonafluoroheptyl)-5-(3-(4-(1,1,3,3,3-pentamethyldisiloxy)butan-1-yl)thiophen-2-yl)thiophene (4d)**: 46% yield as a light yellow oil. ^1^H NMR (400 MHz, CDCl_3_) δ 0.0 (s, 6H), 0.05 (s, 9H), 0.50–0.57 (m, 2H), 1.33–1.43 (m, 2H), 1.58–1.69 (m, 2H), 1.89–1.99 (m, 2H), 2.05–2.21 (m, 2H), 2.69 (t, *J* = 7.3 Hz, 2H), 2.71 (t, *J* = 7.3 Hz, 2H), 6.79 (s, 1H), 6.91 (d, *J* = 5.0 Hz, 1H), 7.17 (d, *J* = 5.0 Hz, 1H); ^13^C{^1^H} NMR (100 MHz, CDCl_3_) δ 0.4, 2.1, 18.3, 20.5 (br), 23.4, 27.4, 29.0, 30.4 (t, *J* = 22 Hz), 34.5, 124.3, 125.1, 126.0, 129.7, 130.2, 133.6, 137.6, 140.3; ^19^F NMR (376 MHz, C_6_F_6_) δ −129.2, −127.6, −117.6, −84.2; IR (ATR): 2957, 1252, 1232, 1167, 1133, 1101, 1057, 1013, 879, 842, 807, 784, 752, 736, 719, 689, 651 cm^−1^; HRMS (DART-ESI^+^) *m*/*z*: calcd for C_24_H_33_^35^ClF_9_OS_2_Si_2_, 663.1056; found, 663.1050.

**2-Chloro-3-(4,4,5,5,6,6,7,7,7-nonafluoroheptyl)-5-(3-(4-(bis(trimethylsiloxy)(methyl)silyl)butan-1-yl)thiophen-2-yl)thiophene (4e)**: 50% yield as a light yellow oil. ^1^H NMR (400 MHz, CDCl_3_) δ 0.0 (s, 3H), 0.07 (s, 18H), 0.44–0.52 (m, 2H), 1.32–1.43 (m, 2H), 1.58–1.68 (m, 2H), 1.89–1.99 (m, 2H), 2.04–2.21 (m, 2H), 2.68 (t, *J* = 7.8 Hz, 2H), 2.70 (t, *J* = 7.8 Hz, 2H), 6.79 (s, 1H), 6.91 (d, *J* = 5.0 Hz, 1H), 7.17 (d, *J* = 5.0 Hz, 1H); ^13^C{^1^H} NMR (100 MHz, CDCl_3_) δ −0.2, 2.0, 17.6, 20.5 (br), 23.1, 27.4, 29.0, 30.4 (t, *J* = 22 Hz), 34.3, 124.3, 125.1, 126.0, 129.7, 130.2, 133.6, 137.6, 140.2; ^19^F NMR (376 MHz, C_6_F_6_) δ −129.2, −127.6, −117.6, −84.2; IR (ATR): 2958, 1252, 1233, 1167, 1134, 1046, 870, 841, 800, 783, 754, 719, 689, 651 cm^−1^; HRMS (DART-ESI^+^) *m*/*z*: calcd for C_27_H_43_^35^ClF_9_OS_2_Si_3_, 737.1608; found, 737.1611.

**General procedure for the polymerization of chlorobithiophene representatives: the reaction of 4b leading to poly(3-(4-(1,1,3,3,3-pentamethyldisiloxy)butan-3-yl)thiophen-2,5-diyl)-*alt*-poly(3-methylthiophen-2,5-diyl) (6b):** To a 20 mL Schlenk tube equipped with a magnetic stirring bar were added **4b** (104 mg, 0.25 mmol) and a 1 M THF solution of TMPMgCl⋅LiCl (0.3 mL, 0.3 mmol) was added at room temperature. After stirring at room temperature for 3 h, THF (2.5 mL) and NiCl_2_(PPh_3_)IPr (**5**, 3.9 mg, 6.0 µmol) were added to initiate the polymerization. The color of the solution changed to light orange and then to dark purple with the formation of slightly insoluble material. After stirring at room temperature for 24 h, the reaction mixture was poured into a mixture of hydrochloric acid (1.0 M, 2 mL) and methanol (10 mL) to form a precipitate, which was filtered off to leave a dark purple solid. After washing with methanol and hexanes repeatedly, the solid was dried under reduced pressure to afford 79.6 mg of **6b** (84% isolated yield). The HT regioregularity was confirmed by ^1^H NMR analysis, and the molecular weight (*M*_n_) and the molecular weight distribution (*M*_w_/*M*_n_) were estimated by SEC analysis. HT = 98%, *M*_n_ = 10400, *M*_w_ = 16900, *M*_w_/*M*_n_ = 1.62; ^1^H NMR (400 MHz, CDCl_3_) δ 0.02–0.08 (br, 15H), 0.50–0.63 (m, 2H), 1.41–1.52 (m, 2H), 1.64–1.78 (m, 2H), 2.44 (s, 3H), 2.75–2.85 (m, 2H), 6.95 (s, 1H), 6.99 (s, 1H); IR (ATR): 2956, 2925, 2855, 1728, 1445, 1252, 1057, 841, 806, 782, 753 cm^−1^.

The other polymers **6c**–**e** were synthesized similarly. The polymerization was continued for 18–24 h or terminated after 1 h when **4** bearing a fluoroalkyl substituent was employed, due to faster polymerization, which brought about an uncontrollable molecular weight. The properties and spectroscopic data are summarized below.

**Poly(3-(4-(bis(trimethylsiloxy)(methyl)silyl)butan-1-yl)thiophen-2,5-diyl)-*alt*-poly(3-methylthiophen-2,5-diyl) (6c)**: 84% yield. *M*_n_ = 70100, *M*_w_/*M*_n_ = 1.38; ^1^H NMR (400 MHz, CDCl_3_) δ 0.03 (s, 3H), 0.09 (s, 18H), 0.43–0.65 (m, 2H), 1.35–1.55 (m, 2H), 1.63–1.85 (m, 2H), 2.44 (s, 3H), 2.71–2.92 (m, 2H), 6.95 (s, 1H), 7.00 (s, 1H); ^13^C{^1^H} NMR (100 MHz, CDCl_3_) δ −0.1, 2.1, 15.9, 17.7, 23.3, 29.4, 34.2, 128.3, 130.0, 130.4, 131.0, 133.5, 134.3, 134.4, 140.0; IR (ATR): 2957, 2926, 2858, 1511, 1449, 1256, 1046, 841, 802, 782, 754, 668 cm^−1^.

**Poly(3-(4-(1,1,3,3,3-pentamethyldisiloxy)butan-3-yl)thiophen-2,5-diyl)-*alt*-poly(3-(4,4,5,5,6,6,7,7,7-nonafluoroheptyl)thiophen-2,5-diyl) (6d)**: 48% yield. *M*_n_ = 68600, *M*_w_/*M*_n_ = 1.15; ^1^H NMR (400 MHz, CDCl_3_) δ 0.06 (s, 15H), 0.55–0.66 (m, 2H), 1.41–1.55 (m, 2H), 1.67–1.83 (m, 2H), 1.97–2.12 (m, 2H), 2.12–2.30 (m, 2H), 2.73–2.88 (m, 2H), 2.88–3.01 (m, 2H), 7.00 (br, 2H); ^13^C{^1^H} NMR (100 MHz, CDCl_3_) δ 0.5, 2.1, 18.4, 21.3 (br), 23.5, 28.7, 29.4, 30.6 (t, *J* = 22 Hz), 34.4, 128.2, 129.3, 130.7, 131.6, 133.3, 134.4, 137.8, 140.4; IR (ATR): 2957, 2925, 1452, 1356, 1252, 1232, 1169, 1134, 1059, 879, 842, 807, 783, 752, 720, 701 cm^−1^.

**Poly(3-(4-(bis(trimethylsiloxy)(methyl)silyl)butan-1-yl)thiophen-2,5-diyl)-*alt*-poly(3-(4,4,5,5,6,6,7,7,7-nonafluoroheptyl)thiophen-2,5-diyl) (6e)**: 58% yield. SEC analysis showed *M*_n_ = 33200, *M*_w_/*M*_n_ = 1.07; ^1^H NMR (400 MHz, CDCl_3_) δ 0.03 (s, 3H), 0.09 (s, 18H), 0.44–0.66 (m, 2H), 1.37–1.60 (m, 2H), 1.60–1.86 (m, 2H), 1.94–2.33 (m, 4H), 2.63–3.11 (m, 4H), 7.00 (br, 2H); ^13^C{^1^H} NMR (100 MHz, CDCl_3_) δ −0.2, 2.0, 17.7, 21.3, 23.3, 28.7, 29.4, 30.6 (t, *J* = 22 Hz), 34.2, 128.2, 129.3, 130.7, 131.6, 133.3, 134.5, 137.8, 140.4; IR (ATR): 2957, 1455, 1356, 1251, 1233, 1134, 1052, 840, 801, 773, 754, 720, 700 cm^−1^.
